# The role of electroencephalography in epilepsy research—From seizures to interictal activity and comorbidities

**DOI:** 10.1111/epi.18282

**Published:** 2025-02-06

**Authors:** Christos Panagiotis Lisgaras, Liset M. de la Prida, Edward Bertram, Mark Cunningham, David Henshall, Anli A. Liu, Vadym Gnatkovsky, Simona Balestrini, Marco de Curtis, Aristea S. Galanopoulou, Julia Jacobs, John G. R. Jefferys, Massimo Mantegazza, Cristina R. Reschke, Premysl Jiruska

**Affiliations:** ^1^ Department of Psychiatry New York University Grossman School of Medicine New York New York USA; ^2^ Center for Dementia Research, The Nathan S. Kline Institute for Psychiatric Research New York State Office of Mental Health Orangeburg New York USA; ^3^ Instituto Cajal CSIC Madrid Spain; ^4^ University of Virginia Charlottesville Virginia USA; ^5^ Discipline of Physiology, School of Medicine Trinity College Dublin Dublin Ireland; ^6^ Department of Physiology and Medical Physics Royal College of Surgeons in Ireland Dublin Ireland; ^7^ FutureNeuro Research Ireland Centre Royal College of Surgeons in Ireland Dublin Ireland; ^8^ Langone Medical Center New York University New York New York USA; ^9^ Department of Neurology, School of Medicine New York University New York New York USA; ^10^ Neuroscience Institute, Langone Medical Center New York University New York New York USA; ^11^ Department of Epileptology University Hospital Bonn (UKB) Bonn Germany; ^12^ Department of Neuroscience and Medical Genetics Meyer Children's Hospital IRCSS Florence Italy; ^13^ University of Florence Florence Italy; ^14^ Department of Clinical & Experimental Epilepsy UCL Queen Square Institute of Neurology London UK; ^15^ Epilepsy Unit Fondazione IRCCS Istituto Neurologico Carlo Besta Milan Italy; ^16^ Saul R. Korey Department of Neurology, Isabelle Rapin Division of Child Neurology Albert Einstein College of Medicine Bronx New York USA; ^17^ Dominick P. Purpura Department of Neuroscience Albert Einstein College of Medicine Bronx New York USA; ^18^ Alberta Children's Hospital Research Institute, Hotchkiss Brain Institute Alberta Health Services & University of Calgary Calgary Canada; ^19^ Department of Physiology, Second Faculty of Medicine Charles University Prague Czech Republic; ^20^ Université Côte d'Azur Valbonne‐Sophia Antipolis France; ^21^ CNRS UMR7275 Institute of Molecular and Cellular Pharmacology (IPMC) Valbonne‐Sophia Antipolis France; ^22^ Inserm U1323 Valbonne‐Sophia Antipolis France; ^23^ School of Pharmacy and Biomolecular Sciences Royal College of Surgeons in Ireland Dublin Ireland

**Keywords:** analysis, animal models, EEG, genetic epilepsies, high‐frequency oscillations, mechanisms, preclinical

## Abstract

Electroencephalography (EEG) has been instrumental in epilepsy research for the past century, both for basic and translational studies. Its contributions have advanced our understanding of epilepsy, shedding light on the pathophysiology and functional organization of epileptic networks, and the mechanisms underlying seizures. Here we re‐examine the historical significance, ongoing relevance, and future trajectories of EEG in epilepsy research. We describe traditional approaches to record brain electrical activity and discuss novel cutting‐edge, large‐scale techniques using micro‐electrode arrays. Contemporary EEG studies explore brain potentials beyond the traditional Berger frequencies to uncover underexplored mechanisms operating at ultra‐slow and high frequencies, which have proven valuable in understanding the principles of ictogenesis, epileptogenesis, and endogenous epileptogenicity. Integrating EEG with modern techniques such as optogenetics, chemogenetics, and imaging provides a more comprehensive understanding of epilepsy. EEG has become an integral element in a powerful suite of tools for capturing epileptic network dynamics across various temporal and spatial scales, ranging from rapid pathological synchronization to the long‐term processes of epileptogenesis or seizure cycles. Advancements in EEG recording techniques parallel the application of sophisticated mathematical analyses and algorithms, significantly augmenting the information yield of EEG recordings. Beyond seizures and interictal activity, EEG has been instrumental in elucidating the mechanisms underlying epilepsy‐related cognitive deficits and other comorbidities. Although EEG remains a cornerstone in epilepsy research, persistent challenges such as limited spatial resolution, artifacts, and the difficulty of long‐term recording highlight the ongoing need for refinement. Despite these challenges, EEG continues to be a fundamental research tool, playing a central role in unraveling disease mechanisms and drug discovery.

**Key points**

- Electroencephalography (EEG) was, is, and will be a fundamental tool in basic and translational epilepsy research.
- EEG in animal models provides useful information that expands well beyond seizure detection.
- The application of EEG techniques to humans will expand due to rapid advances in material, hardware, and software technology.
- EEG alone or combined with imaging, optogenetics, and other modern research tools became a powerful technique for dissecting the cellular and network mechanisms of epilepsies.
- Artificial intelligence in EEG analyses, if used in a manner that is validated by expert EEG readers, can potentially increase its information yield.

## 1 INTRODUCTION

Electroencephalography (EEG) has been a key research technique since the inception of epilepsy research more than a century ago. The first records of utilizing recording of brain electrical activity date to the end of the 19^th^ and the middle of the 20^th^ centuries.^1^, ^2^ With technological advancements in amplifier design, followed by digitization and the utilization of computer analysis, the information extracted from EEG recordings expanded very rapidly. These advances rendered EEG a valuable tool in experimental and clinical epileptology. Throughout this review, we use the term EEG to refer to local field potential (LFP) as well as EEG. However, we recognize that they are not the same.

Since the early studies, a dichotomy in experimental and clinical EEG recording has occurred. Although clinical EEG often captures traditional Berger's frequency bands, experimental research expanded to recordings of brain activities over wide frequency bands ranging from ultra‐slow^3^ to very high frequencies of oscillations.^4^, ^5^, ^6^ It should be noted, however, that aperiodic or non‐rhythmic patterns are also important aspects of EEG, and that they may influence the interpretation of oscillatory (periodic) signals.^7^ Later, new electrode designs were introduced into research including the implementation of multichannel recording,^8^, ^9^ which started to provide more complex information about the spatial topography of epileptiform activity and seizures.^10^ The introduction of new materials and state‐of‐the‐art technologies contributed to the development of new electrodes (injectable, dissolvable, and foldable) with integrated circuits that allow for chronic recording of multichannel population activity combined with recording activity from individual neurons.^10^ Modern epilepsy research utilizes recording techniques that can provide highly complex information about brain dynamics at multiple temporal and spatial scales (discussed in Section 2). The introduction of computers represented a crucial step in epilepsy research. Mathematical analyses of EEG signals dramatically increased the information yield far beyond the visual inspection and became an integral part of brain research. Analytical tools such as advanced machine learning toolboxes brought detailed information about the epileptic network organization, synchronization mechanisms, and underlying dynamical processes.^11^, ^12^

The electrographic phenomena recorded in the epileptic brain are considered a hallmark epilepsy.^13^ For instance, these phenomena include various forms of interictal epileptiform discharges (IEDs), high‐frequency oscillations (HFOs), and other events such as delta brush patterns. EEG studies brought significant insights into the cellular and network mechanisms of epileptogenesis,^14^, ^15^, ^16^ ictogenesis,^17^, ^18^ functional organization of epileptogenic tissues,^19^ and comorbidities.^20^, ^21^ Historically, seizures and IEDs represent key phenomena that pinpoint the presence and activity of epileptic neurons.^13^, ^22^ The introduction of new research techniques such as *in vivo* imaging, optogenetics, and chemogenetics provides novel information about the cellular, metabolic, or vascular contributors to epilepsy. These techniques are often implemented in combination with EEG, and observed phenomena are typically interpreted with respect to the traditional electrographic hallmarks of epilepsy.

In epilepsy research, EEG is typically recorded in small animals (mainly in rodents) using electrodes with diameters ranging from a few micrometers to dozens of micrometers. In humans, this would typically correspond to micro‐EEG.^23^, ^24^ Several types of electrodes can be used to record population activity from individual neurons residing in the vicinity of the electrode as well as extracellular field potentials produced by many neurons, the so‐called LFPs.^9^, ^25^

## 2 WHAT EEG CAN TELL US ABOUT THE BRAIN

Various active neuronal (cellular) processes generate electrical currents across the cellular membranes. The superimposition of these membrane currents in the extracellular space generates an electric field that can be recorded with extracellularly positioned electrodes.^25^, ^26^, ^27^ The major source of EEG is synaptic activity on the dendrites of pyramidal cells. Indeed, when a group of neurons are activated synchronously, the summation of extracellular currents gives rise to electric fields, which can be detectable on EEG.^25^ Both excitatory and inhibitory postsynaptic currents contribute to the EEG signal, where morphology, amplitude, and duration of EEG waveforms depend on factors including but not limited to the number of activated synapses, the number of synchronously activated neurons, and electric field orientation with respect to the electrode.^6^, ^26^, ^27^ Physiological cellular processes involved in functions such as cognition, memory formation, or sleep generate coordinated neuronal activity and membrane potential changes that contribute to information processing and manifest in extracellular recording as electric field fluctuation and various rhythms or oscillations.^28^, ^29^, ^30^, ^31^, ^32^ In epilepsy, the synchronous synaptic activity of large neuronal ensembles and membrane depolarization (e.g., giant excitatory postsynaptic potentials or paroxysmal depolarizing shifts) with strong transmembrane currents manifests as interictal or ictal epileptiform discharges of various morphologies.^13^, ^33^, ^34^ Interictally, the transient excitation is curtailed by subsequent inhibition that correlates with a slow wave on EEG. The cellular and network mechanisms revealed by the study of EEG signals are rich and beyond the scope of this article.^13^, ^35^, ^36^, ^37^, ^38^ Apart from excitatory and inhibitory synaptic activity, other cellular activities contribute to EEG. Large synchronous neuronal depolarizations due to the opening of voltage‐gated channels create depolarizing envelopes (e.g., a burst of action potential riding on dendritic calcium spikes) and massive transmembrane currents as well as synchronous spike‐induced after‐hyperpolarizations due to potassium channel opening.^25^, ^39^, ^40^, ^41^ Extracellular correlates of action potentials of individual cells (single‐unit activity) or multiple cells (multi‐unit activity) can be recorded with special electrodes.^9^ Due to their short duration (<1 ms), neuronal action potential firing can be recorded in LFPs under conditions of very high synchrony. Synchronous action potential (spontaneous or stimulation‐induced) firing manifests on EEG as a deflection designated as population spikes lasting a few milliseconds, which can be observed during seizures or interictal states where they manifest as HFOs—an episode of repeated population spikes.^22^, ^42^, ^43^, ^44^, ^45^, ^46^ In the majority of cases, extracellular field potentials are generated by pyramidal cells (i.e., layer 5 pyramidal cells).^47^ Interneurons contribute to EEG signals largely indirectly through the inhibitory post‐synaptic currents generated on the principal cells or through the modulation of principal cell excitability. However, it should be noted that interneurons can exert a similar contribution to the EEG much like that of big pyramidal cells.^48^ For instance, hilar interneurons contribute directly to the generation of distinct EEG events such as the dentate spike.^49^ Moreover, inhibition mediated by interneurons can orchestrate important functional roles related to memory^50^ and place cell formation^51^ in the hippocampus. In the context of epilepsy models, several experimental preparations have described IEDs generated by the prominent activation of interneurons.^37^, ^52^ Glial cells also influence EEG signals indirectly, apart from ultra‐slow potentials and direct current (DC) potentials, where glial depolarization can contribute directly to EEG genesis.

DC, ultra‐slow, or steady potentials refer to a wide frequency range of brain activities (<0.5 Hz) that are recorded under physiological and pathological conditions. Although experimental studies pay attention to DC recording, DC potentials were neglected in clinical studies for a long time.^6^ It should be noted however that ultra‐slow or DC recordings require specialized equipment such as DC amplifiers and non‐polarizing electrodes.^53^, ^54^, ^55^ DC potentials comprise slow and faster activities corresponding to fast potential fluctuations on EEG. Several pathological activities, such as seizure‐related DC shifts or spreading depolarization, manifest as dramatic changes in DC potential.^56^ DC potentials reflect long‐lasting neuronal and glial depolarizations, which generate large transmembrane currents and are accompanied by significant changes in extracellular potassium^57^ and calcium^58^ concentration. The DC potentials and EEG activity provide comprehensive information about the cellular and network processes that cannot be derived solely from recording traditional EEG activity per se.^59^

Because intracellular studies are often difficult to conduct, extracellular recordings have been valuable in allowing interrogation of a myriad of neuronal and non‐neuronal processes, ranging from cellular firing to network activity at a local or global brain level. Nevertheless, intracellular approaches have become increasingly feasible even in freely moving rodents.^60^ Such novel approaches may significantly expand the use of intracellular approaches that historically have been instrumental in providing direct cellular evidence of the potential origins of EEG waves, including in epilepsy research.^61^

Several processes relevant to epilepsy can be studied at the sub‐millisecond temporal scale,^35^, ^62^ but also at the temporal scale of weeks or months, and over a wide frequency band.^15^, ^16^, ^63^, ^64^ This makes EEG a very powerful tool to explore the epileptic brain. With EEG, fast processes like ictal synchronization, HFO genesis, and cellular firing during populational activities can be studied with high temporal precision.^35^, ^62^ On the other hand, EEG is an effective tool for studying network reorganization and brain dynamics during epileptogenesis, disease progression, seizure cycles, and response to treatment.^15^, ^65^, ^66^, ^67^

Multimodal recording of preclinical and clinical EEG combined with other modalities such as electrocardiography (ECG), respiration, or electromyography (EMG) is instrumental in studying the systemic impact of epileptic activity on cardiovascular, autonomic, respiratory, and motor functions. For instance, the multimodal recording, combined with DC recordings, is critical in preclinical studies of sudden unexpected death in epilepsy (SUDEP^68^, ^69^), where this methodology has brought seminal insights that disclose the relationship between seizures, spreading depolarization, postictal depression, and their role in ictal apnea, terminal respiratory arrest, or seizure‐related cardiovascular abnormalities.

Animal EEG studies have always played a key role in pharmacological research and drug discovery. Seizures represent the primary major symptom of epilepsy. Currently, EEG can provide the most accurate information about the actual number of seizures, even though its accuracy still depends upon the number and spatial arrangement of electrodes used and seizure types^70^ (focal aware, focal with impaired awareness, or generalized). Many seizures can be electrographic only, or can be accompanied by subtle changes in animal behavior that cannot be detected by visual review of video recordings.^65^, ^66^ Similarly, humans can report only a few seizures, whereas hundreds of electrographic seizures can be identified on EEG with patients not being aware of them.^71^ The efficacy of new drugs or new therapies (e.g., gene therapy, gene modulation, neurostimulation) is determined by their efficacy in reducing or completely abolishing seizures, and thus EEG represents one of the most optimal methods to measure therapeutic efficacy.^72^, ^73^, ^74^, ^75^ A limitation is, however, that the customary EEG studies cannot survey the whole brain and, as a result, some very focal seizures may still remain undetected with the surface EEG, requiring more extensive probing with targeted depth EEG electrodes.

**FIGURE 1:**
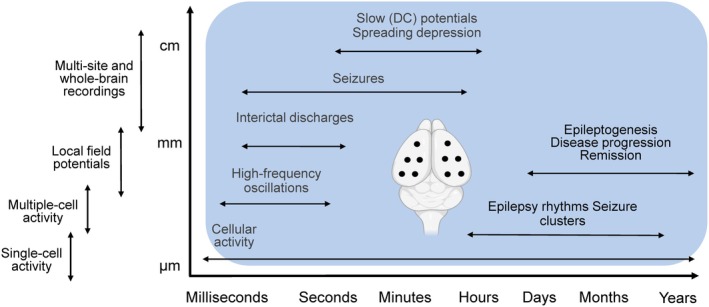
Spatiotemporal properties of experimental electroencephalography (EEG). EEG recorded at a high sampling frequency can explore cerebral activities and processes operating at millisecond or even sub‐millisecond time scales. Therefore, we can study fast processes such as high‐frequency oscillations, interictal discharges, seizure onset, fast propagation, and synchrony. EEG can explore “slow” phenomena operating at the scales of seconds, minutes, or hours—such as seizures, spreading depolarization, or DC shifts. On the contrary, long‐term EEG recording is valuable for studying processes such as circadian rhythms, epilepsy rhythms, epileptogenesis, disease progression, seizure remission, or response to the treatment. The ability of EEG to simultaneously record fast and slow processes is instrumental in studying the long‐term profile of fast activities such as interictal epileptiform activity or high‐frequency oscillations. EEG recording ranging from single to multiple electrodes can provide spatial information from single‐cell activity to nearly a whole brain recording from multiple brain structures. The contemporary state‐of‐the‐art techniques of EEG recording can provide an enormous amount of information about epileptic brain dynamics (a gray area).

Overall, electrophysiological tools enable us to explore brain function at a wide range of spatial and temporal scales (Figure 1) and over a wide range of frequencies, from DC shifts and ultra‐slow fluctuations to high‐frequency activities up to several kilohertz. Therefore, the recording of extracellular activities can provide a quite comprehensive profile of epileptic processes and underlying disease mechanisms.

## 3 THE ROLE OF EEG IN COMORBIDITIES

In addition to epileptic activity, EEG is used to explore, characterize, and monitor physiological or pathological brain signals, activities, or states such as sleep or wakefulness, all of which can directly or indirectly affect cognitive function. Indeed, EEG and extracellular recordings help to disclose neuronal network mechanisms or EEG signals associated with memory and cognitive deficits accompanying epilepsy. For example, various brain oscillations (i.e., theta, alpha, beta, gamma, ripples) may, in certain instances, represent electrographic correlates of highly orchestrated network activity that are instrumental for cognitive function.^32^ Alterations of these rhythms have been described in many experimental models of disease, but a potential link to epilepsy‐related comorbidities requires caution and consideration of the brain area under investigation, age and behavioral state investigated.^76^, ^77^, ^78^, ^79^, ^80^, ^81^ For example, there are certain brain regions such as the hippocampus^82^ that inherently show quite robust oscillations compared to other structures. In this context, brain atlases^83^ describing the distribution of EEG activities or oscillations are valuable to differentiate normal from abnormal EEG rhythms.^80^, ^84^ Moreover, it should be noted that temporal coordination is another critical aspect of brain communication that can be affected in epilepsy and thus contribute to cognitive deficits (for a review, see Ref^85^).

Alterations in cognitive processes can manifest in EEG as a loss of physiological oscillations or changes in their properties, such as changes in the frequency, coupling or amplitude of the oscillation.^86^, ^87^, ^88^ In this context, epileptiform activity is known to disrupt normal oscillations such as those occurring in theta frequency.^81^, ^89^ Sporadic epileptiform activity can impact neuronal processing locally but also in remote areas where it propagates. Indeed, IEDs can exert widespread effects during non–rapid eye movement sleep^90^ that appear to disrupt the large‐scale, cortico‐cortical coupling necessary for normal brain development and cognitive function.^91^

The occurrence of IEDs during a cognitive task can disrupt memory in rats^92^ as well as humans.^93^ Moreover, the frequency of IEDs can predict the degree of neuropsychological impairment. As an example, generalized IED frequency is inversely correlated with performance on generalized intelligence tests and academic performance.^94^, ^95^ Children with idiopathic generalized epilepsies who present with IEDs have deficits in processing speed, attention, visuospatial function, and arithmetic.^96^ Moreover, focal IEDs relate to anatomically specific dysfunction.^97^, ^98^ Scalp EEG and intracranial EEG recordings during continuous cognitive tasks can quantify the dynamic disruption of IEDs. In 1939, Schwab first noticed that “subclinical EEG discharges” slowed reaction time or impaired stimulus response, an electroclinical phenomenon now referred to as transient cognitive impairment.^99^ Of interest, cognitive activity can either facilitate^99^, ^100^ or suppress IED frequency.^101^ Aarts et al.^99^ were the first to demonstrate the spatial specificity of focal IEDs. They showed that left‐lateralized IEDs during verbal information processing were associated with increased error rate, whereas right‐lateralized IEDs affected non‐verbal learning.^99^ Intracranial EEG has permitted a more anatomically precise understanding of how IEDs can disrupt specific cognitive functions. For example, IEDs affecting the lateral frontotemporal cortex prolong reaction time during auditory naming tasks and decrease word repetition speed, suggesting that they contribute to the common cognitive complaint of word‐finding difficulty.^102^ IEDs occurring in the left mesial temporal, lateral temporal neocortical, and fusiform gyrus regions during encoding, and outside the seizure‐onset zone (SOZ), reduce the odds of verbal retrieval^103^ by as much as 15% per event. Likewise, hippocampal IEDs occurring outside the SOZ during the encoding and retrieval phase of a face‐profession association task reduce the odds of remembering by 15% and 25%, respectively,^104^ by putatively decreasing sharp‐wave ripple (SPW‐R) frequency. Indeed, hippocampal IEDs during sleep compete with SPW‐Rs, hijacking normal physiological consolidation processes and thereby disrupting memory consolidation.^86^

Underlying disease mechanisms, and epilepsy‐related structural (e.g., cell loss, synaptic reorganization) and functional (e.g., neurotransmission, excitability, firing properties) changes may interfere with physiological network activity and neural processing leading to various forms of neuropsychiatric comorbidities—intellectual disability, memory decline, and autistic phenotype.^105^ Of note, antiseizure medications can have a similar impact on physiological brain functions.^106^, ^107^

EEG also plays a crucial role in understanding underlying contributors to comorbidities in brain disorders that share many similarities with epilepsy, such as Alzheimer's disease (AD). For instance, patients with AD present with epileptiform activity^108^, ^109^ like patients with epilepsy, they also share rhythmopathies of gamma and ripple oscillations, as demonstrated in animal models.^46^, ^79^, ^110^, ^111^, ^112^, ^113^, ^114^ The presence of epileptiform activity and rhythmopathies on EEG has been associated with impaired cognition in both patients with AD as well as animal models.^108^, ^110^, ^111^, ^113^ In this regard, antiseizure medications can be beneficial in patients with epileptiform activity on EEG.^115^ Seizures can also occur in AD,^116^ where they further exacerbate cognitive impairment.^117^ HFOs such as those recorded in the context of epilepsy^42^, ^66^ are another EEG abnormality that has been reported to occur in several AD mouse lines,^46^ although their role in impaired memory in AD remains unknown. Nevertheless, selective disruption of HFOs in epilepsy using closed‐loop optogenetics restores memory,^118^ in line with previous findings showing that pharmacological normalization of abnormal cell firing underlying HFOs can improve memory recall in epileptic rats.^114^

Taken together, EEG and other electrophysiological techniques are highly instrumental in providing insight into the mechanisms of how epilepsy and other brain disorders that share common EEG disturbances with epilepsy affect cognitive processes. Moreover, EEG represents an effective tool for monitoring the impact of treatments for seizures and epileptiform activity on cognitive functions, thereby disclosing possible mechanisms underlying the beneficial effects of treatments on functional outcomes.

## 4 TRADITIONAL AND NEW EEG RECORDING METHODS

**FIGURE 2:**
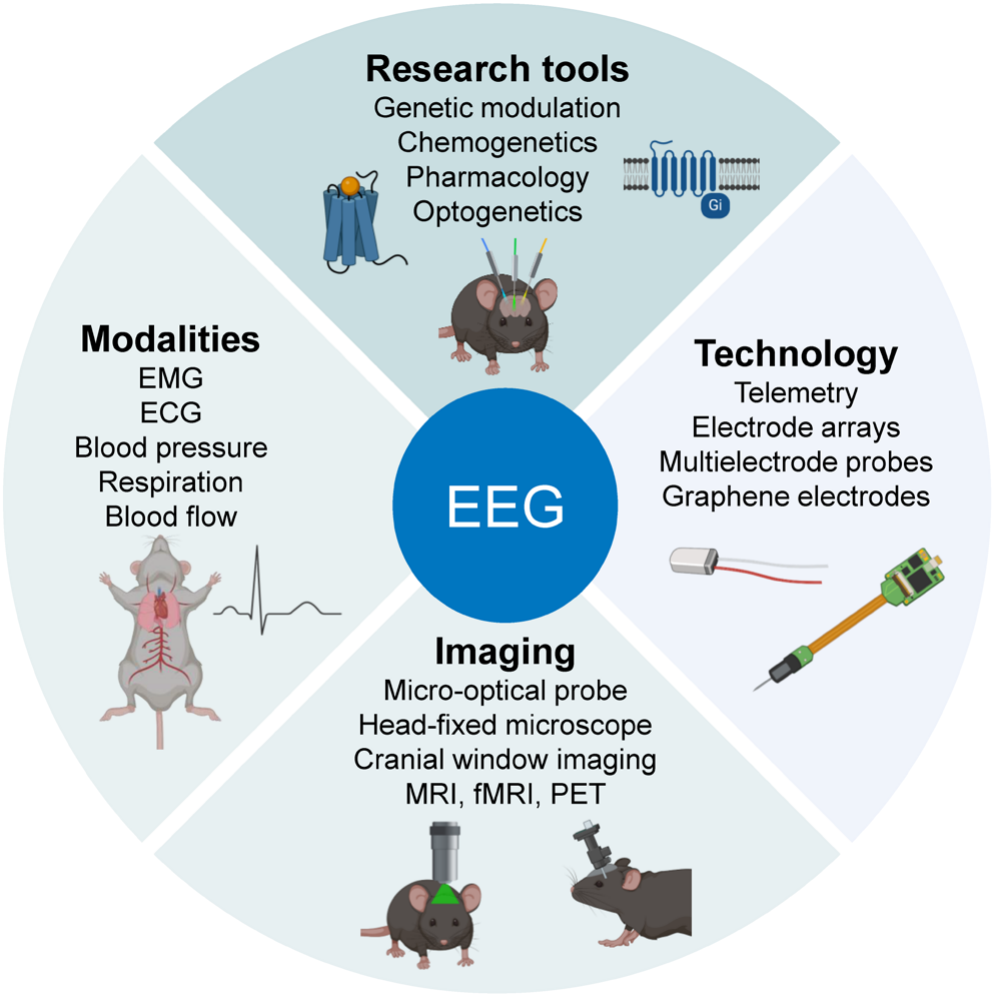
Electroencephalographic (EEG) recording in animals can be combined with other research methods and technologies. EEG can be combined with a variety of *in vivo* structural and functional imaging techniques including a head‐fixed microscope. Apart from brain electrical activity, other physiological parameters such as electrocardiography or respiration activity can be recorded simultaneously with EEG. Technological advances significantly contribute to the improvement of EEG recording techniques including new electrode types and the introduction of novel materials. Modern rechargeable telemetric devices allow for multichannel recordings for nearly unlimited periods of time. EEG is effectively combined with other research tools, such as chemogenetics or optogenetics, to help dissect the network activity with unprecedented cellular specificity. (Created in BioRender.com. Jiruska 2LFUK, P. (2025) https://BioRender.com/q90n176)

The methods of EEG recording in animals became more sophisticated with technological development (Figure 2). Over the last decade, we observed a rapid expansion of advanced methods that allow for long‐term, multi‐site recording of LFPs combined with the recording of unit activity that can provide detailed information about neuronal processes occurring simultaneously across multiple temporal and spatial domains.^10^ Modern epilepsy research benefits from a large portfolio of recording methods, ranging from a simple single EEG electrode to hundreds of EEG channels. Nevertheless, it should be noted that traditional EEG recordings are very often used to validate emerging methods, since they are considered the gold standard in the EEG research arena.

The main principles of traditional animal EEG recording, combined with video monitoring, have been repeatedly described and discussed.^119^, ^120^, ^121^ Nevertheless, one of the most important considerations for selecting the appropriate EEG recording techniques are largely based on the research question(s) and hypotheses we aim to address. One of the first considerations regarding implantation pertains to brain areas of interest. Superficial structures such as the cerebral cortex can be recorded using screw electrodes and modern electrocorticography (ECoG) arrays, whereas deeper structures typically require intracranial approaches using depth electrodes of different sizes, microwires, or micro‐machined electrode assemblies, such as silicon probes. In this context, the choice of wires vs silicon probes depends largely on the spatial resolution one wants to achieve and their budget because silicon probes are typically more expensive.

Two of the most common approaches to record video‐EEG include tethered and radiotelemetry approaches.^119^ Tethered approaches utilize wires that are attached to the subject being recorded, allowing nearly continuous recording over long periods (typically several weeks). Although tethered approaches may restrict the movement of the animal, the use of motorized commutators largely mitigates this effect. In addition, during tethered recordings, animals are typically single‐housed to minimize cable entangling as animals move in the recording chamber, dislodging of the headmounts, or masking an animal's behavior by the cage mate during a target event. However, tethered recordings can limit the ability of animals to interact with littermates socially. In the context of epilepsy, this aspect is of high relevance because single housing has been associated with increased seizure burden.^122^ Thus, telemetry can be advantageous because animal movement is less restrictive compared to tethered approaches. Nowadays, implanted telemetry transmitters have small sizes and record signals from multiple channels at high sampling rates and of various modalities. Even multiple animals with implanted telemetric devices can be housed and recorded together.^123^ Some of the restrictions of telemetry include limited battery life, often limited recording bandwidth, and capacity for much fewer electrodes vs tethered recordings. Nevertheless, some newer developments offer the possibility of wireless charging for telemetry transmitters through inductive charge.^124^, ^125^ Of note, other approaches do not require the use of batteries during recording.^126^ Moreover, in some cases, telemetry devices can be placed on a head mount, making batteries more accessible and easier to replace compared to implantable transmitters. Indeed, this approach has been implemented in seizure‐monitoring experiments performed in non‐human primates.^127^ Other of telemetry are related to the risk of masking animal behaviors from cage mates, signal interruptions or artifacts from cage mates, high cost, and reusability of implants.

In animal models of seizures and epilepsy, advanced electrophysiological methods have been applied to better target the mechanisms and circuits involved, aided by the use of Neuropixel probes,^128^ silicon probe arrays,^129^, ^130^, ^131^, ^132^, ^133^ and tetrodes.^134^, ^135^ Inspired by these data, these tools have been adapted and incorporated into human brain recordings (Table 1). They permit assessing both LFP and unit activity at different temporal and spatial resolutions, which have significantly expanded our understanding of the mechanistic complexities surrounding human brain pathological activity from the cellular level and local networks nearly to whole brain phenomena. In an effort to utilize methods and terms that allow reliable comparison between studies, a series of common data elements (CDEs) have been developed to assist epilepsy researchers.^136^, ^137^ These CDEs are highly useful for experienced neurophysiologists as well as for scientists who would like to introduce EEG into their methodological portfolio.

**TABLE 1:** Advanced electrophysiological methods applied directly to the study of the human brain activity *in vivo*.

Probe design	Description	Types of signals	Reference
Laminar arrays (Ulbert)	Multi‐electrode (24–48 channels) closely spaced arrays (25 μm steps) along a single polyimide penetrating shank (up to 1 mm)	LFP, CSD, and unit activity with laminar resolution	[227]
Grid‐like arrays (Utah)	Two‐dimensional planar (usually 4 × 4 mm) silicon‐based multi‐electrode (currently 96–1024 channels) arrays located at the tip (e.g., 400 μm)	LFP and unit activity at a fixed depth in the cortex. Since the original design, many configurations have been developed, including 3D recordings (Utah slanted design)	[228]
Neuropixels	Ultra‐dense (20 μm spacing) fully integrated linear array (384 user‐selectable channels) distributed along a penetrating shank (10 mm long)	LFP and units all along the probe shank with improved recording stability	[229]
NeuroGrid	Thin‐film‐based (10 × 10 μm) closely spaced array (30 μm) of organic polymer electrodes (256 channels)	Surface electrocorticographic and LFP signals and units	[230]

Abbreviations: 3D, three dimensional; CSD, current source density; LFP, local field potential.

EEG can also be combined with other methods that are currently used in neuroscience research to increase the yield of information, and to dissect cellular and network mechanisms of epileptic activity, including seizures. Thus EEG can be combined with optogenetic stimulation or inhibition to better understand the contribution of different cell types to EEG activity. Optogenetics allows cell‐type–specific control of cellular activity through light delivery. Essentially, the approach requires the expression of genetically engineered, light‐sensitive ion channels or pumps in a tissue of interest and optical control through a light source.^138^ In the context of epilepsy, optogenetic stimulation or inhibition has been deemed a valuable approach to dissecting the role of individual cell types in epileptic network activity or identifying circuit components that can control seizure emergence.^139^, ^140^, ^141^, ^142^, ^143^ Notably, optogenetic manipulations can be tested in real time using closed‐loop systems that are able to detect abnormal epileptic activity in real time, and then trigger light stimulation to inhibit or excite a cell type of interest. Indeed, closed‐loop optogenetics identified circuits with promising seizure‐control capabilities.^118^, ^143^, ^144^, ^145^, ^146^ Moreover, closed‐loop protocols can also integrate electrical stimulation. In those cases, a stimulation electrode is implanted in or near the area of interest,^147^, ^148^, ^149^, ^150^ or stimulation is delivered transcranially.^151^ Some of the advantages of implementing optogenetics over electrical stimulation are related to the cell‐type specificity of the stimulation approach. Nevertheless, off‐target effects of viral targeting should also be considered and controlled. An additional use of optogenetics is related to the development of new models that take advantage of the cell‐type specificity of optogenetics. Thus optokindling^152^, ^153^ has emerged as a complementary approach to the classic kindling paradigm,^154^ where specific cell types are triggered to test the effects of inducing seizures.

Other approaches take advantage of chemogenetic methods^155^ for more prolonged manipulation of neuronal populations, to switch on or off a population of interest and determine whether and how they contribute to (physiological, abnormal, or epileptiform) EEG activity.^156^ A chemogenetic strategy uses a genetically engineered receptor–drug interaction principle and expression of Designer Receptors Activated Only by Designer Drugs (DREADDs) in the tissue of interest.^155^, ^157^ Diverse cell types^133^, ^158^, ^159^, ^160^ have been inhibited or excited to determine their role in hyperexcitability or seizure initiation. One of the advantages of chemogenetic approaches is that prolonged interventions are possible, as the effects of excitation or inhibition can typically last for several hours,^161^ depending on the dose of the compound used to activate the engineered receptor.

Modern *in vivo* imaging techniques combined with EEG recordings have the capacity to provide unprecedented new insights into brain mechanisms in experimental studies.^162^, ^163^, ^164^ This area of neuroscience has expanded rapidly due to the development of genetically engineered indicators and novel microscopic techniques for *in vivo* imaging. A large amount of calcium and voltage indicators are currently available, providing the opportunity to record the activity of brain cells with high cellular and subcellular spatial resolution.^165^, ^166^ Glutamate, γ‐aminobutyric acid (GABA), acetylcholine, potassium, ATP, and other types of indicators allow us to study changes in neurotransmission, neuromodulation, and other molecules during epileptiform activity.^167^, ^168^ However, it should be noted that certain calcium indicators can result in aberrant calcium responses, warranting caution when results are interpreted.^169^ Genetically engineered voltage indicators^170^ represent another approach that offers the ability for one as well as two^171^ photon‐imaging modalities with relatively minimal toxicity.^172^ Moreover, repeated imaging can be performed chronically over the weeks or months through a cranial window or using implantable head‐fixed microscopes. If necessary, deep structures can be reached using implantable micro‐optic fibers. Changes in neuronal or glial activity can thus be monitored over long periods. Long‐term changes in cellular activity and morphology can be correlated with electrographic changes during various stages of epileptogenesis or disease progression. Currently it is not possible to implement such methods in humans. However, future epilepsy research will have the opportunity to utilize these techniques and describe the mechanisms of epilepsy with high precision and resolution that we currently could not achieve by using traditional approaches or analogous recordings in humans.

Overall, given the limitations of each approach, optogenetics, chemogenetics, and *in vivo* imaging studies have greatly contributed to our understanding of the circuits and mechanisms involved in epilepsies. However, further refinement may be necessary to overcome issues related to toxicity and off‐target effects. For example, relatively simple modifications involving the titration of injected virus volume or the use of an alternative promoter could represent effective strategies to address issues related to toxicity.^169^, ^173^ With regard to chemogenetics, the use of alternative ligands^174^ and lower doses^175^ represents approaches to mitigate off‐target effects.

## 5 ADVANCED EEG SIGNAL ANALYSIS

EEG can be assessed visually by experts or, in some applications, audibly after the conversion of EEG signals to sounds. An advantage of visual assessment by experts is that it is based on principles agreed upon by expert clinical neurophysiologists after decades of EEG studies performed with standardized methods. Even though the agreement level in inter‐rater interpretations is not 100%, it provides sufficient power to guide the clinical management of patients or recognize ambiguous EEG signals. Despite its widespread use to study cerebral activity in epilepsy research, it is recognized that the information gleaned from visual inspection can be limited. This limitation arises due to several factors. First, EEG signals are complex, reflecting intricate neural processes involving many different frequencies.^25^ Visual interpretation may overlook subtle patterns, the interaction of such patterns, or nuances that analytical techniques can capture and quantify more effectively. In addition, human interpretation is subjective, time‐consuming, and prone to biases, leading to inconsistencies in findings. Although visual assessment is time‐consuming and requires expertise, it still represents the traditional approach to analyzing EEG. In this context, the principles of EEG interpretation and CDE criteria can be instrumental in EEG data collection and reporting.

Advancements in computer technology and signal processing have enabled the development of sophisticated algorithms for EEG analysis, offering a more comprehensive understanding of brain activity.^176^, ^177^, ^178^, ^179^ Automated methods can detect patterns, anomalies, and correlations that may evade human observation. For instance, automatic approaches with machine learning algorithms can process vast amounts of EEG data, identifying subtle changes with high accuracy.^180^ However, cautious use, including training and validation of such automated approaches in EEG reading by EEG experts, is needed to ascertain the accuracy and biological relevance of data extracted in this manner. Thus, visual assessment remains a valuable tool in EEG analysis. However, the integration of validated automated techniques to enhance diagnostic accuracy and provide more comprehensive insights into brain function and dysfunction is needed.

Several tools are published that provide open‐source platforms for EEG analyses.^181^, ^182^, ^183^ One example is Brainstorm, an open‐source application to analyze various brain recordings such as magnetoencephalography (MEG), EEG, near‐infrared spectroscopy, ECoG, depth electrodes, and multiunit electrophysiology (https://neuroimage.usc.edu/brainstorm/).^184^ *EEGLAB* is another example for processing continuous and event‐related EEG, MEG, and other electrophysiological data.^185^ It integrates independent component analysis (ICA), time‐frequency analysis, artifact rejection, event‐related statistics, and various visualization modes for both averaged and single‐trial data (https://sccn.ucsd.edu/eeglab/index.php). MNE is an open‐source Python package dedicated to exploring, visualizing, and analyzing neurophysiological data.^186^ Other analytical tools can be used for visualizing multimodal data sets containing electrophysiological and behavioral data.^187^, ^188^ In addition, there are several open‐access toolboxes that can be used to analyze epileptiform activity and HFOs, such as RippleLab,^189^ HFOApp,^190^ and PyHFO.^191^ Future developments warrant the sharing and use of open‐access tools to enable cost‐effective analyses and ensure reliable comparison of the results across laboratories.

Classification of the different EEG patterns has been traditionally based on examining characteristics such as their spectral content, morphology, source, state or event dependency and reactivity, as well as the age at appearance.^6^ Extended signal analyses have helped to increase information yield and have provided additional insights into the functional network organization. Measures of synchronization, cross‐frequency coupling, mutual information, and non‐linear interactions have permitted an understanding of the underlying neuronal and network dynamics.^192^, ^193^, ^194^, ^195^, ^196^ Unbiased machine learning and data science tools have revealed a large variety of EEG signatures, which can be explained by different activity inputs and brain‐wide dynamics.^197^, ^198^ These works have demonstrated that latent EEG information can be exploited to derive clinically‐relevant biomarkers.^199^, ^200^ The problem with many of these approaches, however, is the difficulty in understanding how they base their classificatory power. To ease interpretability, some of these studies have used high‐dimensional analysis of the convolutional kernel features^201^, ^202^, ^203^ or recurrent neural networks^204^ to understand what these algorithms recognize as an event.

Application of advanced analytical tools and signal‐processing techniques requires experts with a deep knowledge of mathematics, physics, engineering, computational science, bioinformatics, or complex system dynamics, who have the experience to extract relevant data and provide the appropriate interpretations. It is important to stress that the use of advanced EEG tools in less‐experienced hands or even those with experience may lead to misinterpretation or overinterpretation of EEG data. We recommend caution and consultation with the tools' developers to identify common pitfalls and possible sources of variation in data interpretation. Nevertheless, basic EEG analyses became more accessible to a broader research community through the availability of a wide range of online courses and highly didactic material for non‐experts,^11^, ^205^ interdisciplinary collaboration, and facilitation of programming skills in MATLAB, Python, or R code with tools like ChatGPT.

## 6 CHALLENGES ASSOCIATED WITH EEG AND WAYS TO ADDRESS THEM

Despite EEG having become a sophisticated tool to study epileptic brains, it still has a myriad of limitations. First, it has spatial limitations. Even with many implanted electrodes or multielectrode arrays, the activity is recorded from multiple but spatially limited brain areas and does not cover the whole brain. Moreover, volume conduction can represent an issue when EEG activities are being localized using adjacent electrodes implanted in small rodents like mice or rodent pups. Current‐source density analysis represents a powerful tool to overcome volume conduction issues and identify the true origin of the recorded signals.^25^, ^206^ EEG and LFP recordings rely largely on the difference of potentials between the recording area and the area where the reference electrode is placed, as an absolute electrical reference is absent. Thus, signal interpretation can be challenging, as it may be limited to the reference montage being used.

Animal EEG is an invasive procedure requiring surgery and electrode implantation directly over the cortex or into the brain. As with any surgical procedure, there are risks of surgical complications or confounders that need to be considered, but these procedures are currently required due to a lack of reliable but less invasive alternatives to recording EEG in experimental animals. Recording continuous EEG for extended periods (weeks to months) in developing animals represents a challenge for research exploring developmental or early‐onset epilepsies due to the growing skull and the need for maternal care of developing animals.

Artifacts and EEG interpretations represent persisting and essential limitations of EEG recording.^6^ Although it seems that recording EEG in rodents is a relatively straightforward proposition, EEG interpretation is quite a challenging issue. There are many ways that the method can provide information that does not even originate in the brain but is still misinterpreted, and there are many reports of seizure patterns that are contested and not broadly accepted.^120^ In this regard, the TASK1 group of the International League Against Epilepsy (ILAE)/American Epilepsy Society (AES) Joint Translational Task Force has provided useful reports summarizing the experience in EEG and ECoG studies and interpretation in preclinical studies that can be useful for the reader.^120^, ^176^, ^207^, ^208^, ^209^, ^210^

**FIGURE 3:**
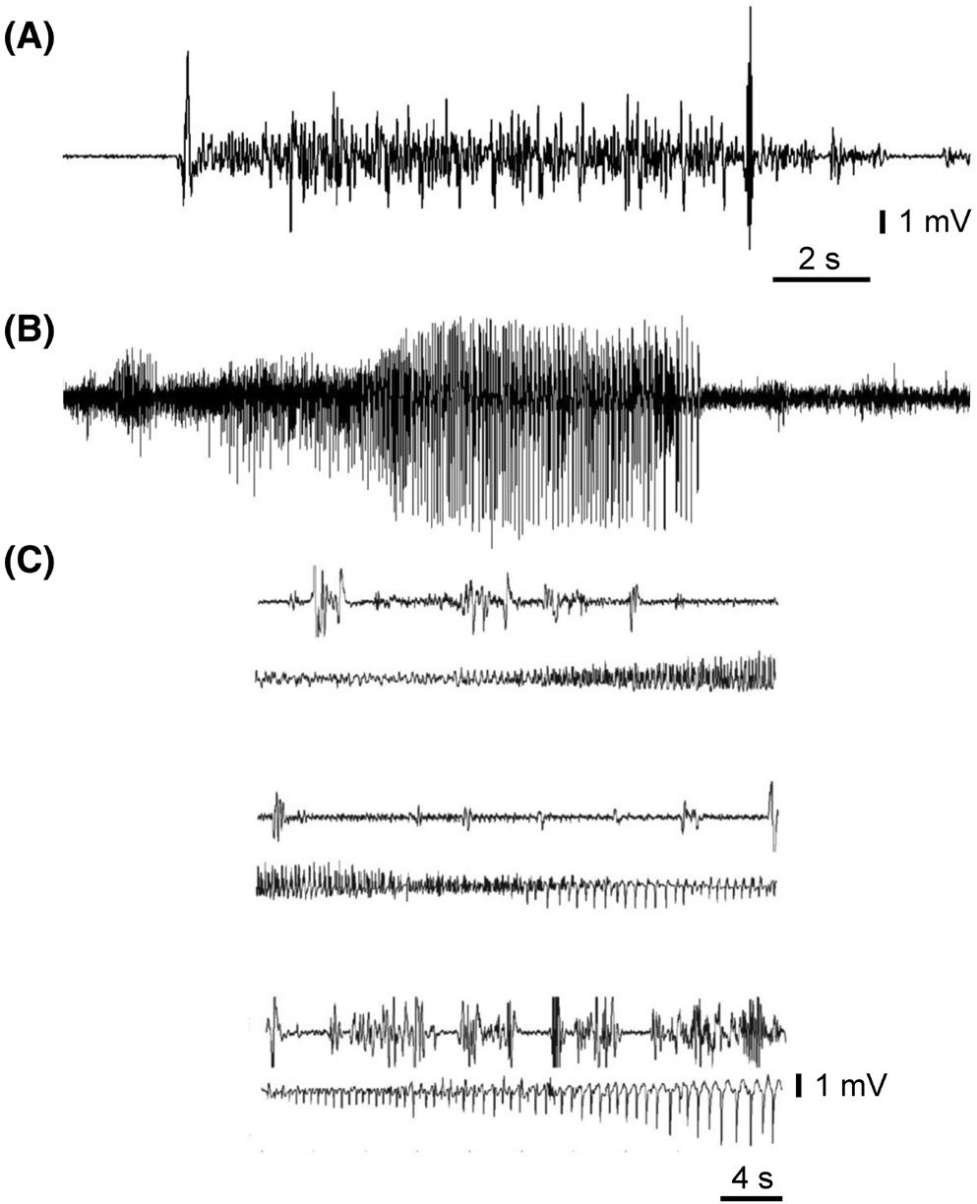
Examples of electroencephalography (EEG) artifacts from animal activity. (A) Movement artifact. Based on a review of simultaneously recorded videos; the rat was quiet and then started moving around the cage. The pattern fits the often‐used criteria for calling an EEG pattern a seizure: At least 5 s of repetitive activity that is at least twice the amplitude of baseline EEG. (B) Head‐scratching artifact. A prevalent pattern that also fits the above seizure criteria and that shows a pattern of evolution over time. As mentioned above, artifact as the source of the EEG change was confirmed by simultaneous video recording. (C) Spontaneous seizure from a rat with limbic epilepsy. Top line is from the frontal cortex; bottom line is from the hippocampus. The high‐amplitude activity in the frontal cortex channel is artifact, mainly from wet dog shakes. The hippocampal channel shows the classic evolution of repetitive activity that changes in amplitude and gradually slows. Of note, the recordings also show that the amplitude of the artifact is often much greater than that of a seizure (5–10 mV or greater vs 2–4 mV in the spontaneous seizure). In addition, the true seizure is much longer in duration (artifacts typically last 10–20 s, and the true seizure 30 s or more).

There are several sources of EEG artifacts.^6^ Artifacts in EEG recordings can arise from extracerebral sources and are a significant limitation in experimental as well as clinical research. They reduce recording quality, obscure cerebral activity, and complicate analysis, and can lead to false interpretations. Every EEG recording, whether clinical or experimental, contains artifacts, which are classified as physiologic or non‐physiologic. Physiologic artifacts, like those from animal movement or muscle activity (EMG), are common because they originate from the biological properties of the subject being monitored. Activities such as head scratching, grooming, or eating generate electric fields that can manifest as high‐amplitude slow or rhythmic EEG activity, closely mimicking seizure patterns (Figure 3). EMG artifacts, typically arising from muscles near electrodes, can obscure EEG signals and are particularly prevalent during wakefulness. High‐quality EEG recordings, with minimal extracerebral interference, are often best obtained during sleep or resting states. Techniques such as bipolar recording, filtering, and shielding can help reduce artifact amplitude. Non‐physiologic artifacts originate from the recording setup, including issues with electrodes, cables, and the recording environment. Electrode‐related artifacts can result from poor contact, degradation, or disconnection, whereas environmental factors like the presence of electric devices (e.g., fridges, mobile phones) can introduce noise. Long cables, damaged connections, and mains supply noise (50/60 Hz) are common non‐physiologic artifacts. Proper setup can minimize these artifacts, including shielding, grounding, and cable management. Removing artifacts is critical for accurate EEG analysis. Although filtering can remove some high‐frequency and slow artifacts, it is not always practical, as it may also eliminate frequencies of cerebral origin. Advanced techniques, such as adaptive artifact rejection and ICA, have been developed to remove specific artifacts while preserving brain signals. In experimental research, ensuring high‐quality equipment and minimizing artifact sources are essential for reliable EEG data.

Correct interpretation represents the next big issue in rodent EEG recording, especially the interpretation of whether the observed pattern represents a seizure. Although definitions between researchers vary, many definitions in the literature range from “repetitive activity of 5 Hz or greater that is at least double the baseline signal amplitude” or “trains of repetitive spikes of at least 5 seconds.” These definitions are easy to follow and automate for quantifying seizures, but validation by experts is needed alongside video records to exclude the possibility that the observed pattern relates to artifactual events. Similarities between artifacts and epileptiform activity also represent a major challenge, even for sophisticated automatic seizure or epileptiform activity detectors. Therefore, it is essential that the investigator verifies that the pattern of interest is not an artifact and uses appropriate controls as a comparison. The issue of appropriate control is important in light of the many transgenic animals now available, as the test animal may have an EEG pattern that is not seen in the control “parent” line that is rhythmic or “spikey” but not a seizure.

One of the issues for those less familiar with EEG is what an EEG represents, especially from intracranial and intracerebral recordings. There is often the sense that we are observing brain activity at a near‐microscopic level, which we are likely not.^6^, ^26^ EEG signals are generated primarily by post‐synaptic potentials on a specific subset of neurons, notably the apical dendrites of pyramidal cells in the cortex or the hippocampus. These extracellular field potentials are best observed when many neurons are synchronously oriented in the same direction, creating what are known as open field potentials, visible from outside their immediate source. Most of this EEG activity originates from thalamic input to these pyramidal cells. In contrast, recording activity from regions with less‐aligned dendrites, like the thalamus or amygdala, requires the electrode to be within the structure, as these non‐laminar fields, known as closed fields, do not project externally.

When interpreting EEG data, it is crucial to recognize these limitations. The signals primarily reflect activity from specific neuron types and regions, whereas significant neuronal activities, especially from local interneurons, may not be captured.^211^ This understanding is essential in avoiding overinterpretation of EEG data, particularly in epilepsy research. Despite its usefulness, the tool has inherent limitations that must be considered for accurate analyses.

## 7 BEYOND THE EEG: PERSPECTIVES AND FUTURE DIRECTIONS

New toolboxes of advanced EEG recording devices enable the examination of brain activity from individual neurons to large‐scale brain‐wide networks (Table 1). In addition, recent developments in graphene‐based arrays enable recording of unit activity, LFP, and DC potentials at unprecedented levels, allowing scientists and clinicians to explore a broader range of EEG frequencies.^212^, ^213^, ^214^, ^215^ Automatic detection of prototypical EEG patterns such as cortical spindles, interictal spikes, and short‐lived HFOs has paved the way for their utilization in clinical practice.^216^, ^217^, ^218^, ^219^, ^220^ More recently, new strategies that leverage machine learning and data science tools have proved transformative for the analysis of EEG. Detection of interictal spikes has benefited from the application of unsupervised data‐driven classification with decision trees, support vector machines, and other criteria without relying on a priori definitions.^221^, ^222^ Artificial neural networks such as convolutional or recurrent neural networks have permitted supervised training for the identification of generically defined oscillations, such as SPW‐Rs in the rodent^203^, ^223^ and primate brain^183^ or predicting seizure patterns.^204^

Other approaches that do not involve the use of video‐EEG are also useful in epilepsy research. One approach uses machine learning–based behavioral phenotyping of mice^224^ to determine hidden behavioral patterns and their evolution during epileptogenesis as well as the response to antiseizure medications. Such an approach has the advantage of monitoring several behavioral signs, which can be mined and used for fast and automated drug screening in the context of epilepsy. Another approach involves the assessment of regional blood flow using functional ultrasound imaging techniques to allow an understanding of the relationship between regional cerebral blood volume (CBV) changes and graphene‐based electrophysiological recordings of epileptic activity.^225^ The advantage of this approach is that unlike functional magnetic resonance imaging, which permits an indirect measurement of blood flow, concurrent electrophysiological recordings are not influenced by magnetic fields. This work has demonstrated that CBV alterations precede seizures and that imaging of blood flow could be a surrogate biomarker to be incorporated in seizure‐detection algorithms.

Undoubtedly EEG has played an essential role in understanding the mechanisms of epilepsy in the last century. A large number of different ictal and interictal epileptiform activities were identified using EEG, which, nowadays, represent gold standard markers that help to diagnose epilepsy, define epileptic syndromes, and localize epileptic tissue clinically and experimentally. Multiple seizure‐onset patterns have been recognized,^43^, ^66^, ^226^ and now we better understand their candidate mechanisms as well as the mechanisms of various forms of interictal activities. The era of EEG is not over—rather the opposite. We expect that EEG recording technology will significantly advance in the near future. New research techniques such as *in vivo* imaging, genetic control of neuronal activity, and multichannel recordings will contribute to the generation of innovative data about the pathogenesis of epilepsy, but they will always be interpreted with respect to simultaneously recorded EEG to allow for informed interpretations. Technological advances introduce new and more sophisticated electrophysiological approaches to record EEG, LFP, and cell activity across multiple temporal, spatial, and frequency scales to provide much more compelling information about the complex nature of epileptic processes. Future methods of EEG analysis implementing artificial intelligence will increase the information yield obtained from EEG recordings and may become even more robust against artifacts. Recently, we have witnessed the expansion of *in vitro* techniques (cell cultures or organoids) to study epilepsy. Unfortunately, these techniques cannot sufficiently grasp the entire complexity and connectivity of the whole rodent or human brain. Circadian rhythms, sleep, long‐term seizure‐risk fluctuation, and complex network interactions within the whole‐brain connectome may be challenging to model and studied using isolated brain tissue. Animal EEG still has a strong position in contemporary epilepsy research, and we predict that this position will not change soon.

## FUNDING INFORMATION

C.P.L. is supported by the National Institute on Aging (R21AG086880) and an NYU FACES Pilot Research Grant. This study was also supported by grants from the Czech Science Foundation (21‐17564S), the Ministry of Health of the Czech Republic (NU21‐08‐00533), the Ministry of Education, Youth and Sports of the Czech Republic (EU—Next Generation EU: LX22NPO5107, ERDF‐Project Brain dynamics CZ.02.01.01/00/22_008/0004643), Medical Research Council UK (MR/X004317/1), Science Foundation Ireland (SFI) (under Grant Number 20/FFP‐P/8613 [Frontier for the Future project award] and Grant Number 16/RC/3948), and the Charles University project EXCITE (UNCE24/MED/021). CURE Epilepsy Cameron Boyce Foundation Taking Flight Award by the Citizens United for Research in Epilepsy, doing business as CURE Epilepsy, Research Ireland under Grant Number 21/RC/10294_P2 and co‐funded under the European Regional Development Fund and by FutureNeuro industry partners to C.R.R. Fundación La Caixa (LCF/PR/HR21/52410030 and LCF/PR/HR22/52420005) to L.M.P. A.S.G. acknowledges grant support from the National Institutes of Health (U54 NS100064, R01NS127524), the U.S. Department of Defense (W81XWH‐22‐1‐0510, W81XWH‐22‐1‐0210), an American Epilepsy Society seed grant, a pilot grant from the NICHD (P50 HD105352) for the Rose F. Kennedy Intellectual and Developmental Disabilities Research Center, the Heffer Family and the Segal Family Foundations, and the Abbe Goldstein/Joshua Lurie and Laurie Marsh/Dan Levitz families. S.B. acknowledges grant support from the National Brain Appeal (NBA), Dravet Syndrome Foundation (DSF), and the Italian Ministry of Health and Tuscany Region (grant code RF‐2021‐12372804, 2023–2026). M.M. acknowledges grant support from the Investissements d'Avenir‐Laboratory of Excellence *Ion Channel Science and Therapeutics* (LabEx ICST ANR‐11‐LABX‐0015‐01) and University Côte d'Azur (IDEX Jedi ANR‐15‐IDEX‐01).

## CONFLICT OF INTEREST STATEMENT

C.P.L., M.O.C., M.dC., M.M., S.B., C.R.R., and P.J. have no conflicts of interest related to the manuscript topic. A.S.G. is the Editor‐in‐Chief of *Epilepsia Open* and associate editor of *Neurobiology of Disease*, and receives royalties from Elsevier, Wolters Kluwer, and MedLink for publications, but has no conflicts of interest associated with this article. We confirm that we have read the Journal's position on issues involved in ethical publication and affirm that this report is consistent with those guidelines.

## Data Availability

Data sharing not applicable to this article as no datasets were generated or analysed during the current study.
